# Aggregates of Chemically Functionalized Multiwalled Carbon Nanotubes as Viscosity Reducers

**DOI:** 10.3390/ma7043251

**Published:** 2014-04-22

**Authors:** Angelo Petriccione, Mauro Zarrelli, Vincenza Antonucci, Michele Giordano

**Affiliations:** 1 Department of Electrical Engineering and Information Technology, University of Naples “Federico II”—DIETI, P.le Tecchio 80, Naples 80100, Italy; E-Mail: angelo.petriccione@unina.it; 2 IPCB-CNR National Research Council—Institute for Polymers, Composites and Biomaterials, P.le E. Fermi, Portici, Naples 80055, Italy; E-Mails: vinanton@unina.it (V.A.); gmichele@unina.it (M.G.)

**Keywords:** viscosity reducers, critical volume fraction, functionalized, nanotubes

## Abstract

Confinement and surface effects provided by nanoparticles have been shown to produce changes in polymer molecules affecting their macroscopic viscosity. Nanoparticles may induce rearrangements in polymer conformation with an increase in free volume significantly lowering the viscosity. This phenomenon is generally attributed to the selective adsorption of the polymer high molar mass fraction onto nanoparticles surface when the polymer radius of gyration is comparable to the nanoparticles characteristic dimensions. Carbon nanotubes seem to be the ideal candidate to induce viscosity reduction of polymer due to both their high surface-to-volume ratio and their nanometric sizes, comparable to the gyration radius of polymer chains. However, the amount of nanotube in a polymer system is limited by the percolation threshold as, above this limit, the formation of a nanotubes network hinders the viscosity reduction effect. Based on these findings, we have used multiwalled carbon nanotubes MWCNT “aggregates” as viscosity reducers. Our results reveal both that the use of nanotube clusters reduce significantly the viscosity of the final system and strongly increase the nanotube limiting concentration for viscosity hindering. By using hydroxyl and carboxyl functionalized nanotubes, this effect has been rather maximized likely due to the hydrogen bridged stabilization of nanotube aggregates.

## 1. Introduction

Polymer compounding is one of the most used way to obtain plastics which ensure processing and performances demands from markets and industries. Polymer-nanocomposites obtained by dispersing organic or inorganic nanoparticles in thermoplastic or thermosetting polymeric matrices nowadays are widely studied to improve structural and functional performances [1–11].

The extremely small sizes of nanoparticles often lead to a contribution to the bulk properties which cannot be explained by classical theories, such as the non-Einstein-like anomalous bulk viscosity of polymer-nanocomposite melts. The first evidence of this phenomenon came from the experimental results reported by Mackay and coworkers [12].

The viscosity of particulate suspensions usually increases with the particle volume fraction, as demonstrated in various experimental and theoretical studies [13–15]. At low volume fractions and for Newtonian fluids as hosting media, interactions among particles can be neglected and the increased bulk viscosity is predictable according to Einstein relation. This relation was progressively extended for non-dilute rigid-sphere suspensions, non-rigid (deformable) and non-spherical particles [16], fiber suspensions [17] and in the case of interacting particles [15]. Metzner *et al*. [18] have reviewed the flow behavior of concentrated suspensions, providing general predictions of flow when solids are suspended in viscous molten polymers. These authors also extended previous theories to non-Newtonian fluids.

Mackay *et al.* [12] considered an ideal system of polystyrene (PS) nanoparticles dispersed in a linear PS melts, in order to reduce the surface enthalpic interactions. Their work highlighted that bulk viscosity decrease is distinctive feature for such a systems in which constituents interact at nanoscale level. In these cases, the viscosity reduction effect is related to an increase in free volume, as also confirmed by the nanoparticle-induced glass transition temperature decrease and by the polymer configuration rearrangements. Tuteja *et al.* [19,20] have expanded these experimental evidences, by disclosing more specific conditions to achieve a reduction in bulk viscosity. They concluded that the polymer melt must be entangled and that the average separation distance among nanoparticles has to be comparable to the polymer radius of gyration (Rg), under such conditions, nanoparticles can perturb polymer chain configurations [19]. In their later work [20], the same authors demonstrated that viscosity reduction also occurs in multifunctional nanocomposite fullerene-polystyrene. Zhang, Lippits and Rastogi [21] tested dispersions of SWCNTs in ultrahigh molecular weight polyethylene by revealing a similar behavior. They have attributed the decrease in viscosity to the selective interaction among nanotubes and to the higher molar mass fraction of polymeric bulk, which, consequently, leads to a decrease of dynamic viscosity for very low percentage (less than 0.1 wt%) of nanoparticles.

In this work we have used multiwalled carbon nanotube “aggregates” as viscosity reducers in a bi-functional epoxy/ammine system. The effect of the aggregates on viscosity has been measured along the occurring *in-situ* polymerization, which promotes a linearization reaction scheme. The interesting phenomena of viscosity reduction is for the first time investigated for an epoxy-thermoplastic system with the aim to discuss and rationalize it irrespective of network formation.

## 2. Results and Discussion

### 2.1. Rheometric Characterization

**Figure 1.**
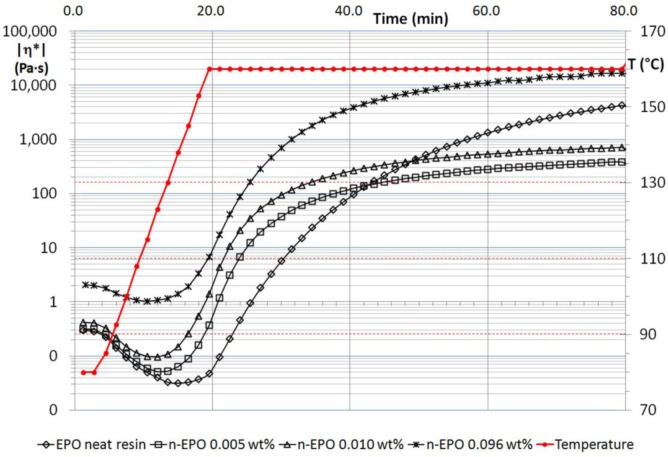
Complex viscosity of n-EPO suspensions compared to neat resin.

**Figure 2.**
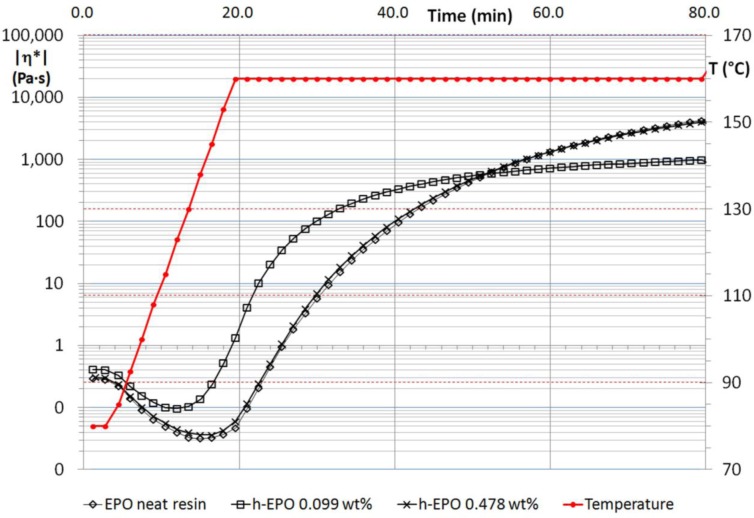
Complex viscosity of h-EPO suspensions compared to neat resin.

**Figure 3.**
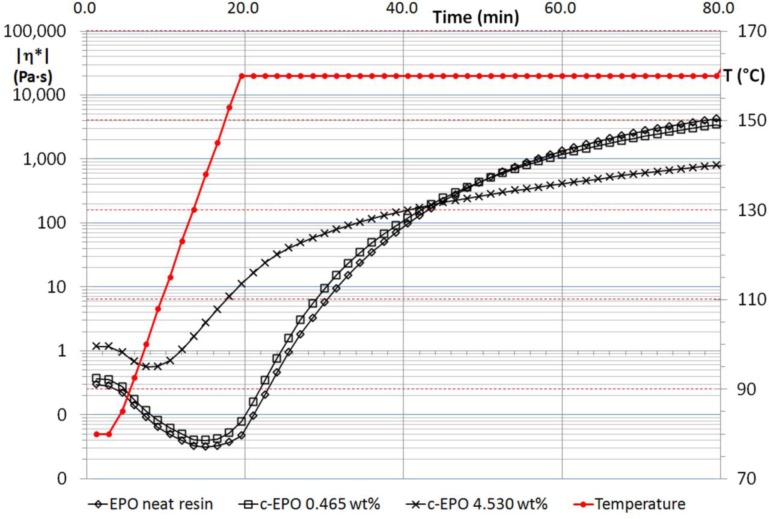
Complex viscosity of c-EPO suspensions compared to neat resin.

MWCNT suspensions, made by un-functionalized and functionalized multiwalled nanotubes, were tested in order to follow changes in rheological behavior during the whole polymerization process. A short nomenclature to classify the specimens has been adopted and hereafter reported with the aim of identifying better the different samples: “n-EPO” refers to the suspensions containing carbon nanotubes without functionalization, “h-EPO” and “c-EPO”, respectively, for suspensions made by –OH and –COOH functionalized nanotubes. Results obtained for n-EPO suspensions have been reported in Figure 1, as well as rheological behavior of neat resin during the whole thermal process. Figures 2 and 3 report, respectively, the corresponding results for h-EPO and c-EPO samples.

Before polymerization the complex viscosity of the n-EPO suspensions are always higher than the corresponding neat resin (see Figure 1). Low molecular weight precursors and nanoparticles interact according to the classical Einstein like behavior, since the complex viscosity of the suspensions increases with arising of the MWCNT content. When polymerization progresses, monomers bond together to form molecular chains with increasing average molecular weight and a radius of gyration Rg. Polymer melts characterized by a lower bulk viscosity than the corresponding neat resin are obtained, according to Tuteja *et al*. [20], for the n-EPO suspensions at respectively 0.005 and 0.010 wt% (nominal values). In these cases, the viscosity values for the polymerized systems @ 160 °C fall down of an order of magnitude compared to the corresponding neat polymer. For n-EPO suspensions, the 0.1wt% concentration of MWCNT is a limiting concentration at which the nanotubes network formation hinders the viscosity reduction effect.

For suspensions made by functionalized MWCNTs (h-EPO and c-EPO), an analogous effect is revealed. All suspensions with ~0.1 wt% nanoparticles exhibit reduced viscosity if compared to n-EPO samples, being the same the amount of nanofiller; h-EPO suspensions with 0.478 wt% of nanotubes reveals, almost the same viscosity of neat polymer (see Figure 2). This later result could be mainly associated to both the improved interface nanoparticle/polymer, which is ensured by the presence of hydroxyl groups on both molecules and by the forming nanoparticle clusterings, which prevent an early percolation as revealed also by the optical characterization reported in the following paragraph.

Similarly to h-EPO specimens, c-EPO suspensions at 0.465 wt% preserve the same viscosity of neat polymer. The rationale for this effect is likely associated with higher nanoparticle content which determines a further effect. In fact, it is believed that functional groups of nanoparticles strongly interact with polymer precursors during the chemical reactions and, also due to the higher amount of this functionalized nanofiller, they can alter the local stoichiometric balance of the reaction preventing the final product of reaching the same average molecular weight.

**Figure 4.**
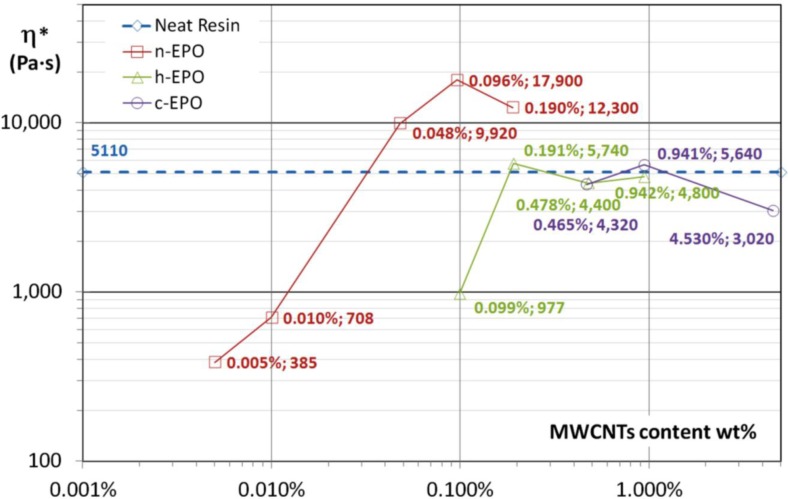
Plateau complex viscosities. Comparison between pristine and functionalized MWCNTs at various weight contents.

In Figure 4, the values of plateau viscosity for all suspensions are reported. Excluding the behavior of c-EPO suspension with ~4.5 wt% MWCNTs content, which could not be compared reasonably to the neat resin and the other specimens, due to stochiometric changes induced by the functional groups, all the other results reveals a very interesting correlation between nanotubes content and plateau viscosity of the polymerized systems. Moreover, it results that functionalization of nanoparticles is very important leading to an huge decrease in viscosity being constant the nanoparticle amount.

### 2.2. Nanoparticles Dispersion: Microscopy

**Figure 5.**
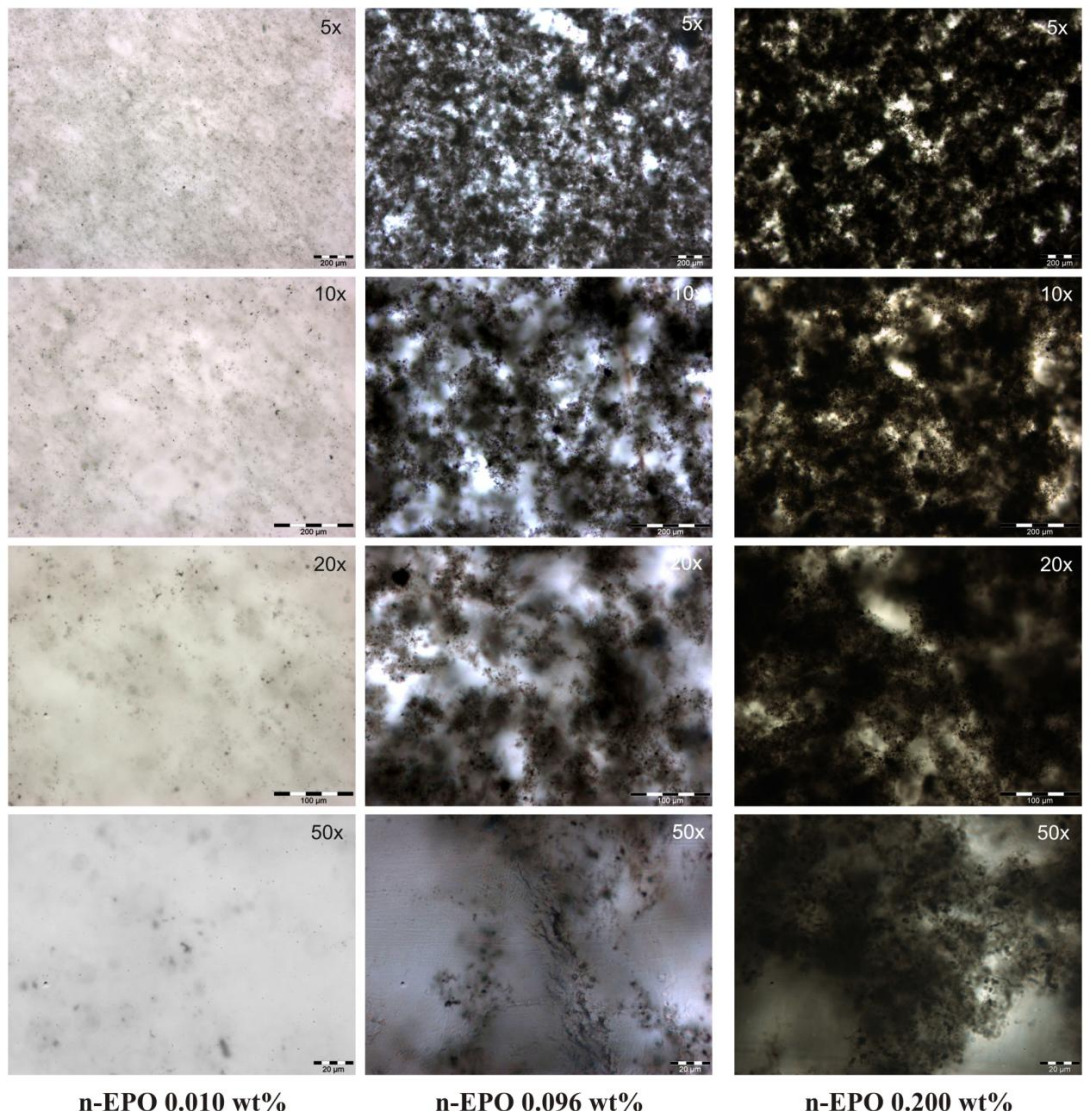
Optical microscopy images of n-EPO samples.

Dispersion and aggregation arrangements can be effectively analyzed at microlevel by using optical microscopy. For n-EPO specimens, an efficient dispersions was achieved with the formation of a fine percolated network at very low concentration of about 0.096 wt% (see Figure 5).

This result matches also with rheological tests (see Figure 1): at this concentration, nanotubes form a percolative network which hinders the effect of viscosity reduction due to the interactions between nanoparticles and polymer chains. At lower concentration levels, the percolation will not occur thus the reduction of system viscosity can be clearly recorded by rheometry.

**Figure 6.**
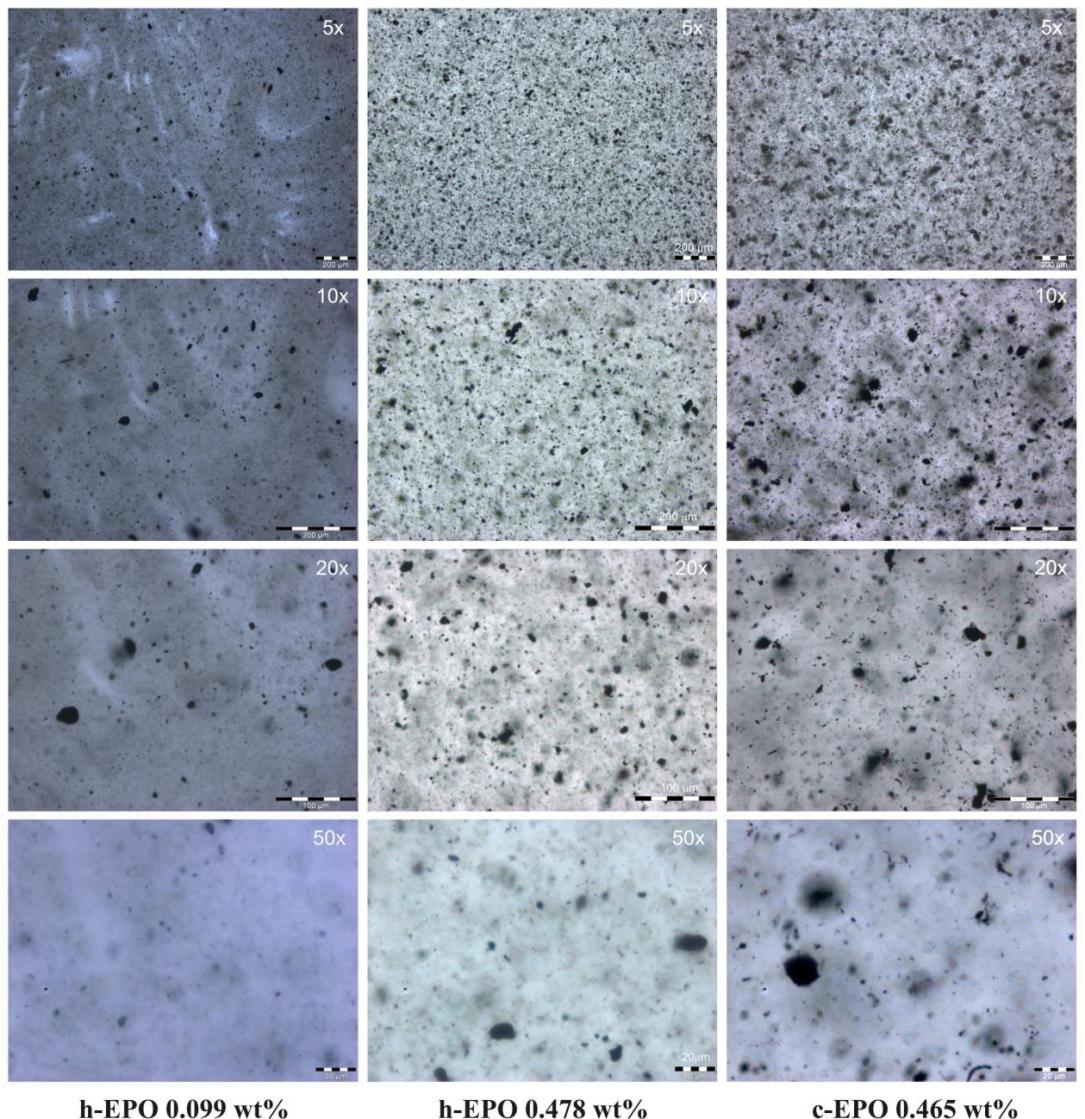
Optical microscopy images of h-EPO and c-EPO samples.

In the case of specimens loaded by functionalized nanoparticles (respectively, h-EPO and c-EPO) optical microscopy analysis has revealed again a good level of achieved dispersion but conversely, the indented structure of nanoparticles network is replaced by a clustered isolated morphology, which prevents percolation (see Figure 6). In order to highlight, how the different dispersion morphologies can affect the rheological behavior, the optical images for n-EPO 0.096 wt% and h-EPO 0.099 wt% specimens could be compared and analyzed. The amount of nanoparticles added is the same (within the experimental error range) however their effect on viscosity results very remarkable with over an order of magnitude difference (see Figure 4).

### 2.3. Nanocomposite Glass Transition Temperature

A further parameter which can be used to investigate effectively the interaction between nanoparticles and polymer matrix is represented by the glass transition temperature of the final system.

**Figure 7.**
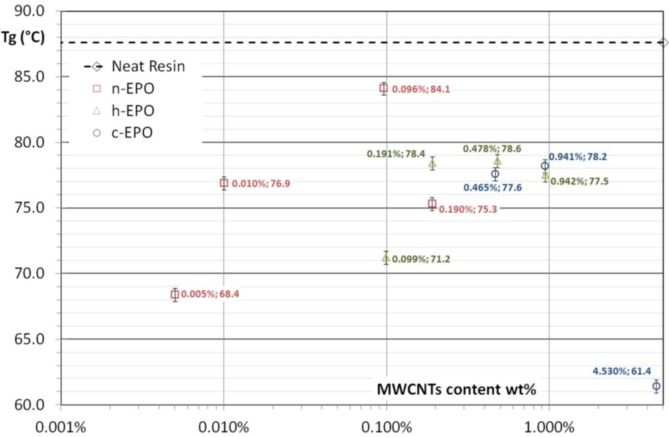
Glass transition temperature for prepared nanocomposites compared to neat polymer Tg.

As glass transition temperature is strictly related with polymer molecular mobility, the final results, obtained for all prepared samples, state that Tg values are lower than the corresponding value for the neat resin system and this arises as an interesting feature for the polymer molecular mobility (see Figure 7).

In fact, lower glass transition levels can be attributed to a higher mobility level of polymer chains. Therefore, a drop of the system viscosity is expected due to the interaction between nanoparticles and polymer structures. On the other hand, by using a higher nanotubes concentration, the complex viscosity increases compared to neat polymer system, and this could be suitably related to the elastic component of the forming percolative network.

## 3. Experimental Section

Raw Materials: the thermoplastic epoxy system was provided by ELANTAS Italia S.r.l. (Parma, Italy). This system is based on diglycidyl ether of bisphenol A (DGEBA) with a low average epoxide equivalent weight (EEW) and abi-functional co-monomers with hydroxyl reactive groups in terminal positions. The supplied material consists of a mixture of the two components without catalyst, separately prepared by melting alchidic ammonium/phosphonium salts in liquid DGEBA, which works as compatible medium with reactive mixture. Epoxy resin and co-reactive agent (polyphenols) have been premixed in quasi-stoichiometric ratio while catalytic paste is added to suspensions until an effective amount of 1 wt% is reached.

Three types of nanotubes have been used in this work. Catalytic carbon vapor deposition (CCVD) grown MWCNTs, namely NC 7000, with an average diameter of 9.5 nm, an average length of 1.5 μm and a purity of 90% were purchased by Nanocyl S.A. (Sambreville, Belgium); –COOH functionalized MWCNT, labelled NC 3151, with an average diameter of 9.5 nm, a length of less than 1 μm, a purity exceeding 95% and a carboxylic functionalization of about 4% purchased by Nanocyl S.A.; –OH functionalized MWCNT, labelled TN-MH3, with a diameter between 10 and 20 nm, a length between 10 and 30 μm, a purity exceeding 95% were purchased by Chengdu Organic Chemicals Co. Ltd. (Chengdu, China), Chinese Academy of Sciences. All nanotubes have been used as received without any extra purification treatment and they have been dried at 110 °C for 6 h at 10 kPa absolute pressure. The addition of functionalized nanotubes in the reactive mixture has been neglected in terms of stoichiometric unbalance; therefore, no correction has been done for the component stoichiometric ratios.

Production of MWCNT-Epoxy suspensions: MWCNT-Epoxy suspensions have been produced using an IKA Ultra-Turrax T25 batch shear mixer (Senaco, Milano, Italy). Due to the high rotation speed of the rotor, the medium to be processed is drawn axially into the dispersion head and then forced radially through the slots in the rotor/stator gap. The dispersion efficiency is comparable to other dispersion techniques like sonication and three-roll milling and is, also, fully scalable for industrial applications.

Optical characterization of polymerized specimens: optical microscopy analysis was carried out on 250 μm thick samples of polymerized suspensions in light transmission mode using an Olympus BX51 microscope (Olympus, Münster, Germany).

Rheologic characterization: Rheological test have been performed on the epoxy suspensions using a test configuration able to measure viscoelastic behavior before and during and after polymerization stage [22–24].Upon preliminary tests on the reactive epoxy system, an oscillatory test has been set up, providing the monitoring of suspension rheological behavior before and during the polymerization stage by measuring variations of characteristic parameters (complex viscosity, G’,G”) of several decades. The oscillatory test has been performed at a constant angular frequency of 5 rad·s^−1^ and a strain amplitude of 10%, ensuring the measure to be conducted in the linear viscoelastic region and the oscillation is sufficiently weak to prevent the nanotube network destruction. The rheological measurements have been carried out under a controlled heating stage where the suspension viscosity has been measured at a constant temperature of 80 °C and during curing stages (ramp at 3 °C·min^−1^ up to 160 °C and holding at 160 °C). The used rheometer is an Anton Paar Physica MCR310 (Anton Paar Gmbh, Graz, Austria), equipped with a Peltier hot/cold stage, which ensures a very effective temperature control (±0.1 °C compliance to setpoint and absence of under/overshoots during transients).

## 4. Conclusions

Multiwalled carbon nanotubes effectively operate as viscosity reducers for polymer matrices, due to both their nanometric sizes, comparable to the gyration radius of polymer chains, and their high surface-to-volume ratio associated with their aggregates morphology.

Clustering of nanotube plays a twofold effect, primarily triggering a selective absorption of high molecular mass polymer chains and, secondarily, increasing the nanotube concentration to achieve “aggregates” percolative network.

Early percolation for unfunctionalized nanotubes hinders the viscosity reduction effects due to the presence of the nanoparticles network. Moreover, it has been found that functionalized nanotubes form hydrogen bridged stabilized aggregates by using hydroxyl and carboxyl groups, which prevent percolation and extend the viscosity reduction effect up to nanoparticle concentrations of about one order of magnitude higher than the corresponding threshold for unfunctionalized carbon nanotubes.
